# Early enteral vs. oral nutrition after Whipple procedure: Study protocol for a multicentric randomized controlled trial (NUTRIWHI trial)

**DOI:** 10.3389/fonc.2022.855784

**Published:** 2022-06-28

**Authors:** Gaëtan-Romain Joliat, David Martin, Ismail Labgaa, Emmanuel Melloul, Emilie Uldry, Nermin Halkic, Ginette Fotsing, Alessandra Cristaudi, Pietro Majno-Hurst, Dionisios Vrochides, Nicolas Demartines, Markus Schäfer

**Affiliations:** ^1^ Department of Visceral Surgery, Lausanne University Hospital CHUV, University of Lausanne (UNIL), Lausanne, Switzerland; ^2^ Graduate School for Health Sciences, University of Bern, Bern, Switzerland; ^3^ Department of Endocrinology, Diabetology and Metabolism, Lausanne University Hospital CHUV, Lausanne, Switzerland; ^4^ Department of Surgery, Regional Hospital of Lugano, Lugano, Switzerland; ^5^ Division of Hepatobiliary and Pancreatic Surgery, Carolinas Medical Center, Charlotte, NC, United States

**Keywords:** pancreas cancer, pancreatoduodenectomy, malnutrition, complications, morbidity

## Abstract

**Background:**

Malnutrition has been shown to be a risk factor for postoperative complications after pancreatoduodenectomy (PD). In addition, patients needing a PD, such as patients with pancreatic cancer or chronic pancreatitis, often are malnourished. The best route of postoperative nutrition after PD remains unknown. The aim of this randomized controlled trial is to evaluate if early postoperative enteral nutrition can decrease complications after PD compared to oral nutrition.

**Methods:**

This multicenter, open-label, randomized controlled trial will include 128 patients undergoing PD with a nutritional risk screening ≥3. Patients will be randomized 1:1 using variable block randomization stratified by center to receive either early enteral nutrition (intervention group) or oral nutrition (control group) after PD. Patients in the intervention group will receive enteral nutrition since the first night of the operation (250 ml/12 h), and enteral nutrition will be increased daily if tolerated until 1000 ml/12 h. The primary outcome will be the Comprehensive Complication Index (CCI) at 90 days after PD.

**Discussion:**

This study with its multicentric and randomized design will permit to establish if early postoperative enteral nutrition after PD improves postoperative outcomes compared to oral nutrition in malnourished patients.

**Clinical trial registration:**

https://clinicaltrials.gov/(NCT05042882) Registration date: September 2021.

## Introduction

Pancreatic ductal adenocarcinoma (PDAC) is one of the deadliest cancers in humans (more than 47’000 estimated deaths in 2020 in the United States) (1). It is predicted to become the second most common cause of cancer deaths in the United States by 2030 (2). The mean costs in 2015 were estimated to be $79’800 per patient with PDAC and $164’100 for each resection (3). The observed overall 3-year survival after diagnosis is 6% (4, 5). Surgery remains the only potentially curative strategy when combined with adjuvant or neoadjuvant chemotherapy. However, resection of the pancreatic head remains a difficult surgical procedure with high morbidity (40-60%) (6, 7). Recently, the concept of Enhanced Recovery After Surgery (ERAS) has contributed to reduce overall morbidity, length of hospital stay and costs by implementing multimodal measures influencing the pre-, intra- and postoperative periods (8–12).

Patients suffering from pancreatic tumors as well as patients with chronic pancreatitis often present with cachexia or at least with a certain level of malnutrition (13). This situation is difficult to correct preoperatively. Nutritional therapy should therefore be started early during the postoperative course to prevent further malnutrition, as the latter is an important risk factor to develop complications (14–16). In addition, surgery disrupts the digestive tract, leading to postoperative indigestion and malabsorption (17). Postoperative nutritional supports, including early enteral nutrition (EEN) and parenteral nutrition (PN), have been shown to be effective in improving clinical outcomes after major abdominal surgery (14).

Malnutrition is still poorly defined. Many definitions have been proposed based on criteria that vary between medical history, biometric and biological data. Currently, the European Society for Parenteral and Enteral Nutrition (ESPEN) recommends the Nutritional Risk Score (NRS) as a screening tool, even if it has not been prospectively validated (18, 19). Several studies have proven its reliability to identify patients at nutritional risk who will benefit from perioperative nutritional support (15, 20). Patients with NRS ≥3 are considered to be exposed to higher incidence and severity of postoperative complications.

Recent randomized clinical trials and meta-analyses have shown that EEN could shorten length of stay, reduce postoperative infections and mortality and improve cost-effectiveness when compared to PN in gastrointestinal cancer surgery (21–24). Specifically after pancreatoduodenectomy (PD), EEN has been shown in one study to reduce early and late complications, infections, and readmission rates (25). Another retrospective study showed no differences with respect to time to resumption of normal oral intake, morbidity and mortality when comparing EEN *via* nasojejunal tube or jejunostomy tube and parenteral nutrition (26). However, a recent multicentric randomized controlled trial that compared nasojejunal EEN to PN after PD showed that EEN was associated with an increase of overall postoperative complications (27). One major drawback of this study is that it did not compare EEN to the recognized standard which is oral feeding and not PN (28). Another systematic review compared the outcomes of 5 feeding routes after PD (oral diet, enteral nutrition *via* either a nasojejunal, gastrojejunostomy tube or jejunostomy tube, and PN) and reported no evidence to support routine enteral or parenteral feeding after PD (29).

The study of EEN and its impact in terms of morbidity require the use of a validated tool. Most studies fail to provide information about the severity of complications and inform only on the most severe event, ignoring events of lesser severity (30). The Comprehensive Complication Index (CCI) was created to summarize all postoperative complications and is more sensitive than existing morbidity endpoints (31).

The primary objective of the study is to assess the impact of EEN through a jejunal tube placed intraoperatively on postoperative morbidity after PD, according to the CCI. Secondary objectives are to assess the impact of EEN on major postoperative complications, according to Clavien classification, specific complications, length of stay, readmission rates, reoperations, quality of life (QoL), metabolic stress and nutritional response after PD.

## Materials and methods

### Hypothesis and primary/secondary objectives

The hypothesis is that EEN after PD might decrease the postoperative complications compared to oral nutrition as patients undergoing PD often are malnourished. The primary objective is to assess the impact of EEN on postoperative morbidity after PD, according to the CCI, in patients at nutritional risk with a NRS ≥3.

The secondary objective is to evaluate the impact of EEN on major postoperative complications, according to Clavien classification (defined as ≥3a), specific complications, length of stay, readmission rates, reoperations, QoL, metabolic stress and nutritional response after PD in patients at nutritional risk with a NRS ≥3.

#### Primary and secondary endpoints

The primary endpoint measuring postoperative morbidity will be assessed using the CCI at 90 postoperative days.

Secondary endpoints are the following:

- Most severe postoperative complications (≥3a) will be measured using the Clavien classification within 90 postoperative days.
- Specific complications of PD will be recorded:
  ◯ Surgical site infections (SSI), further divided into ‘superficial’, ‘deep’ and ‘organ-space’ according to the specific anatomic involvement and the Centers for Disease Prevention definition (32).
  ◯ Postoperative pancreatic fistulas (POPF) are classified into three grades, A, B and C, according to the consensus of the International Study Group for Pancreatic Surgery (ISGPS) (33).
  ◯ Delayed gastric emptying (DGE), which is classified into three grades, A, B and C, according to the consensus of the ISGPS (34).
  ◯ Postoperative pancreatic hemorrhage (PPH), which is also classified into three grades, A, B and C, according to the consensus of the ISGPS (35).
  ◯ Biliary fistula (no standard definition)
  ◯ Gastrojejunal anastomosis fistula (no standard definition)
  ◯ Pancreatitis (no standard definition)
- Length of stay will be measured from operative day until discharge.
- Readmissions will be counted until postoperative day 90.
- Reoperations
- Patients’ QoL will be assessed by the EORTC (European Organisation for Research and Treatment of Cancer) QLQ-C30 questionnaire (36). This questionnaire will be filled 4 times: at preoperative consultation or admission, at patient’s discharge, between the 4th and 6th postoperative week and on POD 90 (via phone call).
- The subjective tolerance of EEN will be assessed daily during the first 7 postoperative days, using a visual analogue scale (0: perfect tolerance to 10: no tolerance). Objective tolerance will be assessed by the amount of EEN as a percentage (tolerated/total amount of EEN required).
- Time required (in days) to reach respectively 50% and 100% of the daily caloric targets required (30 kcal/kg/day if BMI <30 kg/m2 and 25 kcal/kg/day if BMI ≥30 kg/m2, protein target: 1.5 g/kg/day).
- Metabolic response to EEN will be assessed with biological measurements preoperatively and twice weekly (currently already measured, according to our PD care map):
  ◯ C-Reactive Protein (CRP) and procalcitonin
  ◯ Simple blood count, coagulation
  ◯ Electrolytes: sodium, potassium, calcium, magnesium, phosphate
  ◯ Creatinine, urea, blood glucose, liver and pancreatic function tests, prealbumin, albumin, triglycerides
- Various malabsorption due to PD surgery: measurements will be made twice, once before surgery and once between the 4th and 6th postoperative week during the follow-up visit. As PD might induce duodenal and pancreatic insufficiencies postoperatively due to the resection of the duodenum and the pancreatic head, it is presently unknown if EEN might influence these insufficiencies by improving the overall nutritional state and the mucosal trophic status of the small bowels.
  ◯ Duodenal insufficiency: folate, magnesium, calcium, iron, ferritin, transferrin saturation
  ◯ Exocrine pancreatic insufficiency: malabsorption of fat-soluble vitamins: vitamin D (with calcium/phosphate balance and parathormone) and vitamin E
  ◯ Endocrine pancreatic insufficiency: due to risk of developing a secondary diabetes, HbA1c (glycated hemoglobin) will be measured.
- Body measure using bioelectrical impedance analysis (BIA) and muscle strength using handgrip will be measured preoperatively, on discharge day, and on the first follow-up visit. BIA will calculate the percentage of body fat and muscle mass using 2 or 4 electrodes on the wrists/fingers and ankles to measure the impedance. Handgrip will be measured in both hands (best of 3 attempts).
- Resting energy expenditure will be measured bedside by the dietician on POD 5 using indirect calorimetry. Indirect calorimetry will measure respiratory gas exchange using a canopy hood or a face mask.

Each of these endpoints will be measured in the study (EEN) and control (oral nutrition) groups.

#### Study design

This study is an open-label, multicentric, international two-arm, randomized controlled trial.

#### Study intervention

After PD, patients will be randomized to receive either EEN or oral nutrition. Patients included in the EEN arm will receive, in addition to oral nutrition based on the current care maps, enteral nutrition according to the following scheme that was established in accordance with nutritionists:

- Six hours after the operation, a low flow enteral feeding will be initiated (21 ml/h, 250 ml/12h), and based on a Isosource^®^ Energy Fibre solution (or similar product, 400 kcal).
- If the tolerance is subjectively good, with a visual analogue scale ≤4/10, the flow will be increased on first postoperative day (POD), at the flow of 42 ml/h (500 ml/12h from 8 pm to 8 am, 800 kcal).
- On POD 2: increased flow to 62.5 ml/h (750 ml/12h, 1200 kcal)
- On POD 3: increased flow to 83.5 ml/h (1000 ml/12h, 1600 kcal)

If the tolerance is not satisfactory (5/10 and 6/10), the current flow will be maintained 24 hours more. It will be decreased to previous stage if tolerance is >6/10 or put on hold for 6 hours in case of persisting digestive symptoms (severe nausea, vomiting, severe bloating or severe diarrhea) despite diminution of the nutrition flow, and increased the next day if tolerated until the maximum of 1000 ml/12h.

The diet will be infused over 12 hours with a pump and controlled flow rate. EEN will be continued until oral food intake will have reached more than 50% of nutritional requirements. Daily nutritional requirements will be defined as 30 kcal/kg if BMI <30 kg/m2 and 25 kcal/kg if BMI ≥30 kg/m2 (protein target: 1.5 g/kg/day). The oral intake will be assessed by the dietitian.

If a patient in the EEN group loses or displaces its nasojejunal tube (vomiting, accidental removal), a new probe will be replaced under endoscopic control by the gastroenterologist through the gastrojejunal anastomosis. If this happens a second time, another attempt to put the nasojejunal tube will not be made (the patient will remain in the study). In the same previous scenario (nasojejunal tube expulsion), and if the patient suffers from DGE, a nasogastric tube will be installed at the same time.

In the enteral nutrition group, if the patient suffers from DGE and the nasojejunal tube is in place, a nasogastric suction tube will be installed in addition, and enteral feeding will be continued. Parenteral nutrition will be used to complete, if necessary, the missing caloric needs. In the control group (without jejunal tube), in case of DGE, a nasogastric suction tube will be installed (the patient will remain in the study) and parenteral nutrition will be started.

The use of parenteral feeding will be standardized similarly in both groups. A parenteral nutrition will be initiated if the caloric intake is <50% of caloric requirements for 24 hours and from POD 3. Parenteral nutrition will be continued until the total caloric intake without the parenteral nutrition reaches >50% of daily caloric needs and until no more nasogastric tube will be in place.

### Inclusion and exclusion criteria, justification of study population

Participants fulfilling all the following inclusion criteria are eligible for the study:

- Patient scheduled for elective open PD.
- Patient ≥18 years old.
- Patient at nutritional risk, i.e., with NRS ≥3.

The presence of any one of the following exclusion criteria will lead to exclusion of the participant:

- Patient not able to give informed consent as documented by signature of consent form (e.g., vulnerable patients).
- Enteral feeding already initiated preoperatively.
- Inability to follow the procedures of the study, e.g., due to language problems, psychological disorders (i.e., eating disorders and bipolar disorders), or dementia.

The total number of included patients will be 128 (64 in each group). The choice of the patient population is justified by the fact the patients undergoing PD often are malnourished (cachexia due to cancer or chronic pancreatitis) and are at nutritional risk postoperatively due to the important stress response induced by this major abdominal surgery. As malnutrition is a risk factor for complications, EEN might reduce the morbidity burden after PD.

#### Recruitment, screening and informed consent procedure

The study will be proposed to any patient planned for a PD meeting inclusion criteria. The study will be presented to the patients during the first preoperative consultation by the investigators at the hospital. Expected benefits (fewer postoperative complications) and potential disadvantages as well as risks (poor tolerance of the nasojejunal tube, nausea, vomiting, diarrhea, tube obstruction, bronchial inhalation) will be explained. An information sheet will be given to the patient during the preoperative consultation. The patient will have the opportunity to ask questions.

A time of reflection will be given (at least 24 hours). The consent form will therefore be obtained at last the day before the intervention.

The investigators will explain to each participant the nature of the study, its purpose, the procedures involved, the expected duration, the potential risks and benefits and any discomfort it may entail. Each participant will be informed that the participation in the study is voluntary and that he or she may withdraw from the study at any time and that withdrawal of consent will not affect his or her subsequent medical assistance and treatment.

The participant will be informed that his or her medical records may be examined by authorized individuals other than their treating physician.

All participants for the study will be provided a participant information sheet and a consent form describing the study and providing sufficient information for participant to make an informed decision about their participation in the study.

The formal consent of a participant, using the approved consent form, will be obtained before the participant is submitted to any study procedure.

The consent form will be signed and dated by the investigator or his designee at the same time as the participant signs. A copy of the signed informed consent will be given to the study participant. The consent form will be retained as part of the study records. The informed consent process will be documented in the patient file and any discrepancy to the process described in this protocol will be explained.

#### Study procedures

Given the rate of annual procedures, a recruitment of about 60% of eligible patients, and based on the experience of two randomized studies successfully completed in the CHUV Visceral Surgery Department (NCT00508300, NCT00512213), the planned overall study duration is three years including the recruitment period and follow-up. For patients the study duration will be from enrolment until POD 90, date of last follow-up phone call. The expected hospitalization duration for each patient will be approximately 14 days, which is the current mean hospital stay after PD in our department.

Eligibility of the patients will be confirmed on the day before the operation (day -1). Then, patients will be randomized before the operation (day -1) or on operation day in either the control arm or the experimental one (EEN).

Standardized surgical procedure in all eligible patients: In terms of surgical details, all patients will receive a prophylactic dose of antibiotics (cefuroxime) 30 minutes before incision and first have exploratory laparotomy followed by conventional or pylorus-preserving PD. Pancreaticojejunostomy will be performed. The technique of the pancreaticojejunal anastomosis will be left to the surgeon choice. End-to-side hepaticojejunostomy will be performed with single-layer interrupted sutures. A gastrojejunostomy on an omega loop will finally be constructed approximately 70 cm distally to the ligament of Treitz. One or two perianastomotic drains will be placed.

Specific study procedure: At the end of surgery (after the three anastomoses are finished but before closure of the abdomen) and in the EEN study group only, a polyurethane single or double lumen feeding nasojejunal tube (Freka^®^) 8F will be inserted by the anesthesiologist and placed under direct palpation and visual control by the surgeon, 30 cm distally to the gastrojejunostomy into the alimentary limb (jejunum). The tube will be attached according to current practice to the nose wing with a tape. The patient will therefore be under general anesthesia during tube insertion and no x-ray control will be needed. An accepted alternative to nasojejunal tube will be to place a surgical gastrojejunal tube at the end of the operation.

Postoperatively, patients will receive standardized perioperative care according to the ERAS protocol in both arms.

From a nutritional point of view, this includes:

- The day before surgery: 2 carbohydrate drinks of 200 ml
- The operative day: 2 carbohydrate drinks of 200 ml up to 2 hours preoperatively, then postoperative free drinks
- On postoperative day (POD) 1: broths, creams, yogurts, drinks ≥2l
- On POD 2: light diet, drinks ≥2l
- On POD 3: normal diet (half serving)
- On POD 4: normal diet (full serving)

From POD 1, patients of both groups will receive two oral nutritional supplements (Resource^®^ Ultra XS 125 ml, 280 kcal, 18 grams of proteins or analogous products) until discharge. In terms of intravenous infusions, a parenteral crystalloid solution will be used (Ringer-Lactate): 1000 ml during operative day and on POD 1, 500 ml during POD 2 and 3, then 250 ml, if necessary, until POD 8 (minimum for maintenance of the central venous line). Anti-nausea agents (ondansetron 4 mg 3x/j and mephameson 4 mg 1x/j) as well as laxatives (magnesium hydroxyde 4.5 g 2x/j) will be used daily for 3 days, then on demand. Prokinetic agent (metoclopramide 10 mg 3x/j) will be used on demand. An anti-acid (esomeprazole 40 mg 1x/j) will be introduced for the duration of the hospitalization. Digestive enzymes will be prescribed from the first postoperative day (Creon 40’000 UI 3x/j). The dose of digestive enzymes will be adapted based on the quantity of oral food that the patient will eat.

In terms of mobilization, patients will be stimulated by nurses/physiotherapists according to the following plan:

- Operative day: just get up from bed
- On POD 1: walk once during the day, spend ≥6h out of bed (3 x 2h)
- On POD 2 to discharge: walk twice during the day, spend ≥ 8h out of bed (4 x 2h)

In our current practice, no suction gastric tube is routinely left in place after the operation, but this choice will be left to the surgeon and recorded in the electronical case report form.

A standard nutrition protocol for the EEN intervention group will be prescribed as established in accordance with the nutritionists. Patients randomized into the oral nutrition group will receive the current postoperative management and receive from POD 1 an oral nutrition that will be gradually increased if tolerated until a normal diet (see above).

Several blood tests will be performed during the postoperative period. A timeline summary table of all study visits, relevant procedures, and samplings is shown in **Table 1** (schedule of assessments).

**Table 1:** Schedule of assessments.

Study periods	Screening	Entry	Intervention			Discharge	Follow-up	
Visits	1	2	Daily			3	4	5
Days	Preoperative	-1	0	1-7	Hospitalization	14^	30-45	90
Patient information and informed consent	x*	x*						
Patient eligibility confirmation		x						
Demographics	x	x						
Randomization		x						
Standard surgery (not study procedure)			x					
Nasojejunal tube (EEN group)			x***					
Physical examination	x	x	Daily			x	x	
Vital signs	x	x	Daily			x	x	
Metabolic tests ¥		x			2x/week			
Nutrition tests Ω		x					x	
Body measures°		x				x	x	
Indirect calorimetry				on POD 5				
EORTC QLQ-C30	x**	x**				x	x	x
Subjective tolerance (VAS 0-10)				x				
Complications - CCI						x	x	x
Complications - Clavien						x	x	x
LOS						x		
Readmissions								x

Demographics include the measure of serum CA 19-9 at admission in case of pancreatic cancer.

EEN, early enteral nutrition; EORTC, European Organisation for Research and Treatment of Cancer; VAS, Visual Analog Scale; CCI, Comprehensive Complication Index; LOS, length of stay; POD, postoperative day.

¥Metabolic tests: CRP, procalcitonin, simple blood count, coagulation tests, electrolytes (sodium, potassium, calcium, magnesium, phosphate), creatinine, urea, blood glucose, liver and pancreatic function tests, prealbumin, albumin, triglycerides.

ΩNutrition tests: folate, magnesium, iron, ferritin, transferrin saturation, vitamin D, calcium, phosphate and parathormone, vitamin E, HbA1c (glycated hemoglobin).

°Body measures: lean body mass using bioelectrical impedance analysis and strength using handgrip.

*Filled either during screening visit or at hospital admission.

**The preoperative EORTC questionnaire will be filled during screening visit or on hospital entry day. In total, four questionnaires will be filled. The last one on postoperative day 90 will be filled by the study nurse who will perform the phone call.

***At the end of surgery and in the EEN study group only: polyurethane single or double lumen feeding nasojejunal tube (Freka®) 8F or surgically-placed gastrojejunal tube.

^Discharge on day 14: current mean hospital stay.

Demographic disparities or differences in patient characteristics could be a source of bias. To reduce this risk, we decided to undertake a randomization of the participants. Moreover, heterogeneity in general management between centers might be a source of bias. Randomization will be stratified by center to decrease the risk of center bias.

#### Withdrawal and discontinuation

Patients will be withdrawn from the study if they leave the operation room with only a suction nasogastric tube in place or in case of withdrawal of informed consent, non-compliance to the study protocol, or due to safety concerns. Participants will not be replaced and considered as dropouts. Study data already collected on a participant until the time of withdrawal will still be used for analysis in a coded manner. No further data will be collected however from that time onwards.

### Assessment

The CCI will be assessed on postoperative day 90. All complications that occurred during the 90 days after PD will be included for each patient in the CCI. The CCI is a global index of postoperative morbidity that includes all the complications that a patient present and is based on the Clavien classification. The CCI is graded from 0 (no complication) to 100 (death) by using an algorithm available online (https://www.assessurgery.com/about_cci-calculator).

### Follow-up

Follow-ups will be performed 4 weeks after hospital discharge with physical consultations and on POD 90 with phone calls.

### Statistical analysis and sample size calculation

A statistician was involved in the study design and estimate of the sample size. A statistician will realize the statistical analyses once all data will have been collected.

Null hypothesis H0: EEN has no effect on postoperative complications (CCI) in the population (and therefore the observed effect is entirely due to chance): p2 = p1.

Scientific hypothesis H1: EEN has an effect on postoperative morbidity (CCI) in the population (and therefore the observed effect is not entirely due to chance): p2 > p1.

According to a previous randomized trial including a series of PD and assessing a realimentation process (enteral *vs*. parenteral), the mean CCI was impacted of about 30% (32.8 *vs*. 24.2) (27). Another study reported a mean CCI of 38 after PD (37).

Based on the above results, we hypothesize that ENN will reduce by 30% a mean CCI of 35 (+/- 20) of the oral nutrition group. We will therefore expect a mean CCI for the treatment group (EEN) of 24.5 (SD 20). In this superiority study, for a power of 80% and a significance level of p-value ≤0.05 (two-sided alpha), we will therefore need 57 patients per group according to the sample size calculation. Nevertheless, we will increase the sample size to a total of 128 patients to take into account 10% of drop-out (e.g., due to discomfort associated with the tube or nasojejunal tube displacement) at 90 days (primary endpoint evaluation). We will therefore need to enroll 64 patients per group in the trial.

The study will be closed once the required 128 patients will be included. No interim analysis will be performed.

Normality of distribution will be determined by the Kolmogorov-Smirnov test and quantile-quantile plots of dependent variables for all continuous variables.

We will use a Student’s *t-*test to evaluate if the primary outcome (CCI) can significantly be reduced by EEN compared to the control group (comparison of mean CCI hypothesizing a normal distribution) if the normality of distribution is confirmed. On the contrary if normality is not satisfied a Mann-Whitney *U* test will be used. For the analysis of all secondary endpoints, we will also use *t-*tests (or Mann-Whitney *U* tests if distribution is not normal) or chi-square tests based on the variable types. Regarding the questionnaires filled 4 times during the study, tests specific to repetitive ordinal measures will be used.

The primary analysis will be based on the intention-to-treat method and not per protocol. We will perform an intention-to-treat analysis so that all patients being intended to treat will be analyzed in the statistics independently. All patients will therefore be analyzed according to the group in which they were initially randomized. The intention-to-treat population (full analysis set) will be defined as the groups of patients who were randomized to have enteral nutrition (intervention group) or oral nutrition (control group). The inclusion in the enteral or oral groups will be defined at the moment of randomization, not taking into account if the patients finally received the specific postoperative nutrition based on the study protocol. As a sensitivity analysis, we will perform a per protocol analysis. The per protocol analysis will permit to assess the effect of enteral nutrition if correctly received as mentioned in the study protocol.

Blocked randomization will be done using a computerized algorithm *via* REDCap by a research coordinator the day before surgery. The proportion of “study” (EEN) and “control” (oral nutrition) subjects will be 1:1 (mix of variable block sizes of 4, 6, and 8 patients, randomly selected). Before surgery, only the responsible surgeon will know the allocation group. Postoperatively, the inclusion in the different groups will be known by the caregiver team and the patient, as it is not possible to blind the intervention (nasojejunal tube). The investigators, the outcome adjudicators, and the data analysts will be blinded (allocation concealment).

The statistical package used for analysis will be SPSS version 26 (IBM Corp., Armonk, NY, USA).

#### Handling of missing data and drop-outs

In case of missing data among variables other than endpoints (adjustment variables, >5% of expected data) we will consider the use of the multiple imputation technique. This process will be performed multiple times (e.g., 10-20 times) to combine multiple data sets to produce one final data sets replacing the missing data (38). A 10% drop-out was considered in the sample size calculation.

## Discussion

### Anticipated results

The hypothesis of this study is that EEN through a jejunal feeding tube will permit to provide to patients the required calories after PD more rapidly and will decrease the number and severity of postoperative complications after PD. The authors anticipate a decrease of the CCI on POD 90 by 30% in the EEN group compared to patients with oral nutrition.

#### Overall ethical considerations

The study design (randomized controlled trial) will permit to have a good internal validity of the study. Moreover, the CCI used as main outcome is a validated index of general postoperative morbidity and it enables to encompass all complications that a patient may present. The inclusion of several centers internationally will increase the generalizability of the results (external validity). The complication rate after PD remains high (around 60%) and malnutrition has been established as a risk factor of postoperative complication. An intervention that could improve the nutritional status of the patients may lead to a decrease of morbidity after PD.

If the results are favorable, this study will permit to establish an EEN protocol to improve patient outcomes after PD. Patients undergoing PD could rapidly benefit of this management, and EEN could become the new standard of care for the perioperative nutrition management after PD. The results of this study could be implemented and translated into daily clinical practice promptly.

The need for research in this field is clearly present, as the issue of postoperative nutrition after PD is not resolved and does not reach a consensus among pancreatic surgeons. The numerous presentations, debates in congresses, and our recently published survey on that subject attest and highlight the absence of consensus and lack of solid data (39).

The results of this study would go beyond the only scientific interest, as they will directly impact patients undergoing pancreas surgery. As pancreas cancer incidence is projected to grow in the upcoming years, pancreas surgery number will correlatively increase. Ultimately, in the current era of growing health expenditures and need for cost containment, if EEN allows decreasing complications and length of stay, it could also decrease the overall costs for each patient hospitalization for PD, which could have important positive repercussions on the health care system.

Particular attention will be paid to the process of randomization to ensure a sound methodology. An overall fair balance for the study participant will be maintained.

#### Risk-benefit assessment

There are potential adverse events associated with nasojejunal tube and enteral nutrition: poor tolerance, nausea, vomiting, diarrhea, tube obstruction, or bronchial inhalation. Nursing teams are trained to use the equipment and will perform the same care as usual: position verification, flushing, nasal fixation, nasal eschar surveillance. With these measures, the risk of adverse events associated with the study is judged to be low. In addition, the intraoperative positioning of the tube in the efferent alimentary loop should minimize these risks.

From the investigators’ perspective, we hypothesize that the EEN intervention will be a benefit for patients included in the study group. Benefits of EEN could be a decrease of postoperative complications and a shorter length of stay. Nevertheless, the control group cannot be considered disadvantaged as the current recommended nutrition management after PD is oral nutrition. It is also possible that the participation to the study will not bring any benefits.

## Conclusion

This study will bring new insights on the impact of enteral nutrition on postoperative complications after PD in malnourished patients compared to oral nutrition.

## Data availability statement

The original data will be included in the future articles and supplementary materials. Further inquiries can be directed to the corresponding author.

## Ethics statement

This study involving human participants was reviewed and approved by the Ethics commission of Vaud, Lausanne, Switzerland (Commission cantonale d’éthique de la recherche sur l’être humain, CER-VD). The patients/participants will provide their written informed consent to participate in this study.

## Author contributions

G-RJ, DM, and MS designed the study. G-RJ drafted the manuscript. All authors contributed to the manuscript. All authors reviewed and approved the final version of the manuscript.

## Funding

This project is supported by the Livio-Glauser Foundation, Lausanne, Switzerland. Open access funding provided by University of Lausanne.

## Conflict of interest

The authors declare that the research was conducted in the absence of any commercial or financial relationships that could be construed as a potential conflict of interest.

## Publisher’s note

All claims expressed in this article are solely those of the authors and do not necessarily represent those of their affiliated organizations, or those of the publisher, the editors and the reviewers. Any product that may be evaluated in this article, or claim that may be made by its manufacturer, is not guaranteed or endorsed by the publisher.
